# PD89 Global Versus Regional Evidence To Evaluate Robotic-Assisted Surgery: A Systematic Literature Review And Meta-Analysis

**DOI:** 10.1017/S026646232510216X

**Published:** 2025-12-29

**Authors:** Ana Yankovsky, Usha Kreaden, April Hebert, Neera Patel, Matthew Lien

## Abstract

**Introduction:**

Da Vinci robot-assisted surgery (dV-RAS) has been around for more than 20 years and has become the standard of care for select procedures in specific countries. Recently, health technology assessment agencies have used local evidence to inform their evaluation of dV-RAS. We aimed to compare global, Pan-European (Pan-EU), and Pan-Asian systematic literature reviews on dV-RAS for seven malignant procedures.

**Methods:**

The PubMed, Scopus, and Embase databases were systematically searched through to 31 December 2022 following PRISMA and PROSPERO guidelines (CRD42023466759). Studies that compared dV-RAS with laparoscopic or video-assisted thoracoscopic surgery (lap/VATS) or open oncologic surgery and reported on relevant clinical outcomes were included. Studies were checked for the source of clinical data and categorized as being either Pan-EU or Pan-Asian. The global analysis included all eligible studies. Pooled odds ratios or mean differences were calculated for randomized, prospective, and database studies using R software and a fixed-effect or random-effects (when heterogeneity was significant) model. The revised Cochrane risk-of-bias and ROBINS-I tools were used to assess bias.

**Results:**

The global systematic literature review identified 230 studies (34 randomized, 74 prospective, 122 database), including 78 Pan-EU and 35 Pan-Asian studies. Results from the global and regional studies agreed on rates of conversions and blood transfusions and lengths of hospital stay. The global, Pan-EU, and Pan-Asian studies did not align on: operative time (Global: dV-RAS longer; Pan-EU: dV-RAS longer versus open; Pan-Asian: dV-RAS longer versus lap/VATS); 30-day complications (Global and Pan-Asian: dV-RAS lower; Pan-EU: dV-RAS lower versus open); 30-day readmissions (Global and Pan-EU: dV-RAS lower; Pan-Asian: dV-RAS lower versus open); 30-day reoperations (Global: dV-RAS lower versus open; Pan-EU and Pan-Asian: dV-RAS similar); 30-day mortality (Global: dV-RAS lower; Pan-EU: dV-RAS lower versus open; Pan-Asian: dV-RAS longer versus lap/VATS).

**Conclusions:**

Our analysis highlighted that global and regional evidence aligned on select outcomes. The differences we observed might be associated with surgeon experience, limited evidence, or other unknown regional biases. These findings should be considered by health technology assessment agencies when looking at regional data versus global evidence.

